# Small agarics in Taiwan: *Mycena albopilosa* sp. nov. and *Gloiocephala epiphylla*

**DOI:** 10.1186/s40529-017-0173-y

**Published:** 2017-04-04

**Authors:** Yi-Yin Chang, Yu-Ming Ju

**Affiliations:** 1 Department of Life Sciences, National Taiwan University, Taipei, Taiwan; 2 Institute of Plant and Microbial Biology, Academia Sinica, Taipei, Taiwan

**Keywords:** Small agarics, Basidiomata production, *Mycena*, *Gloiocephala*, Taiwan

## Abstract

**Background:**

Small agarics are poorly documented in Taiwan, with previously reported species either rudimentarily described or lacking a description or diagnosis in most cases. A survey on small agarics in a lowland forest of Taiwan revealed two species previously unrecorded.

**Results:**

One agaric, which is characterized mainly by white hairs overlying the pileus, a conspicuous cup-shaped basal disc surrounding the stipe, and inamyloid basidiospores, fits the genus *Mycena* and appears undescribed. It readily produced abundant basidiomata in culture in three weeks. The other agaric is *Gloiocephala epiphylla*, being characterized by its reduced hymenium and conspicuous pileogloeocystidia. ITS sequences from the two agarics further corroborated the identifications. Their macroscopic and microscopic features and culture morphology are described. A dichotomous key is provided to the species of *Mycena* reported in Taiwan.

**Conclusion:**

The *Mycena* species is newly described as *M*. *albopilosa* herein. *Gloiocephala epiphylla* is new to Taiwan, being the only species of the genus known on the island.

## Background

Small agarics with a pileus less than 1 cm diam pose a challenge for mycobiotic surveys, because they are easily overlooked and, after collected, their delicate, fragile basidiomata need to be measured and recorded in a timely fashion. Most of these small agarics belong to *Mycena* (Pers.) Roussel*, Marasmius* Fr., and *Galerina* Earle, each of which contains hundreds of taxa. Some of the small agarics can be found in genera such as *Gloiocephala* Massee and *Omphalina* Quél. They are saprophytes or in close association with mosses (Davey et al. 2013), while a few of them have been reported as being mycorrhizal (Zhang et al. 2012) or parasitic (Dennis 1957; Baker and Holliday 1957; Sequeira 1958; Bayliss 1911). These overlooked small agarics are actually underestimated. Notable plant pathogenic species include *Mycena citricolor* (Berk. & M.A. Curtis) Sacc., which causes the well-known American leaf spot that decreased annual yields of coffee crops by 20% (Sequeira 1958; Rao and Tewari 1987), and *Marasmius perniciosus* Stahel, which causes witches’ broom disease on cacao in South America (Baker and Holliday 1957; Evans 1980). Certain *Marasmius* species contain laccases and other enzymes capable of degrading aromatic compounds, lignin, and β-carotene (Dedeyan et al. 2000; Scheibner et al. 2008). Noticeably, more than 30 species of *Mycena* are known bioluminescent (Desjardin et al. 2007).

There are over 500 *Mycena* species described in the world (Desjardin et al. 2008). In Taiwan, 21 *Mycena* species have been reported, but, in most cases, a species is merely furnished with a rudimentary diagnosis or lacks a description/diagnosis entirely. Shih et al. (2014) described a bioluminescent species *M. kentingensis* Shih et al., which represents the only novel species of the genus documented in Taiwan thus far. Despite the great potential and special features that these small agarics may possess, without extraordinary characters such as bioluminescence and pathogenicity, their taxonomy hardly becomes a research interest for mycologists in Taiwan.

In the present study, *Mycena albopilosa*, which is characterized by a white, minute, hairy pileus and a conspicuous cup-shaped basal disc at the stipe, is described as new. It has characteristics of *Mycena* section *Sacchariferae* Kühner ex Sing. in general, but its basidiospores are inamyloid. In addition, *Gloiocephala epiphylla,* the type species of the genus, is reported and represents a newly recorded genus in Taiwan. Both *M. albopilosa* and *G. epiphylla* are tiny, growing on substrates, such as fallen leaves or twigs, in humid forests.

## Methods

### Specimen collecting and culturing

Basidiomata together with the attached substrate were brought back to the laboratory. The substrates were placed in the plastic chambers with high humidity maintained for further basidiomata production. Freshly produced basidiomata were placed onto 2% malt extract agar (MEA) for collecting basidiospores. Germinating basidiospores were transferred to fresh MEA. Cultures were subsequently transferred to potato dextrose agar (PDA), MEA, and oatmeal agar (OA). Growth rate and culture morphology were recorded every 3–5 days.

Both species were also cultured on a formulated soil, which was modified from the one used for *Mycena kentingensis* (Shih et al. 2014), with white rice bran replaced by black rice bran. The formula was as follows: 80% potting soil (Green Orchids Co. #521) thoroughly mixed with 20% black rice bran. The mixture was divided into 10 g per 9-cm Petri dish and autoclaved at 121 °C for 30 min before use. Mycelia were grown in the laboratory at 25 °C for 4 weeks. To maintain the water content, the humidity was kept approximately 50–70%, and 1 ml sterile water was sprayed every 4–7 days on each Petri dish.

### Morphological study

Macro- and micro-morphological data of *Mycena albopilosa* were mainly based on cultured fruiting bodies due to exhaustion of original specimens. The morphology of specimens artificially cultivated was similar to those growing in natural habitat. Color terms and notations used for morphological and culture features were compared with Methuen Hand Book of Colour (Konerup and Wanscher 1978). Microscopic observations of *Gloiocephala epiphylla* were rehydrated with distilled water and stained with Melzer’s reagent. Hyphal tissue organization was observed from resin fixation and sectioning of basidiomata. Basidiospores were measured with a Leica DM2500 microscope under 100× immersion oil objective and 10× ocular. Basidiospore statistics were calculated by Piximetre 5.8, including the arithmetic mean and range of the spore length and spore width (30 spores in a specimen); Q: the range of spore length to width quotient.

### DNA sequencing

Detailed methods were referred to Hsieh et al. (2009). Fungal mycelia were harvested from 1 to 2 weeks culture and transferred into flasks containing malt extract broth, which were incubated in a rotary incubator under 25 °C for 7–10 days. The mycelia were then freeze-dried and stored in a refrigerator at 4 °C. Total DNAs were extracted using a MX-16 automatic nucleic acid extractor (Compacbio Sciences, Burlingame, CA, U.S.A.) with Maxwell^®^ 16 Tissue DNA Purification kit (PROMEGA Corp., Madison, WI, U.S.A.). Polymerase chain reaction amplifications of ribosomal internal transcribed spacers (ITS) were described in detail in Hsieh et al. (2009). The ABI Prism model 3730 × 1 DNA Analyzer^®^ (Applied Biosystems, Foster City, CA, U.S.A.) was used for DNA sequencing.

## Results and discussion

### *Mycena albopilosa* Y.-Y. Chang & Y.-M. Ju, sp. nov., Figs. 1, 2, 3

MycoBank number: MB 815859

**Fig. 1:**
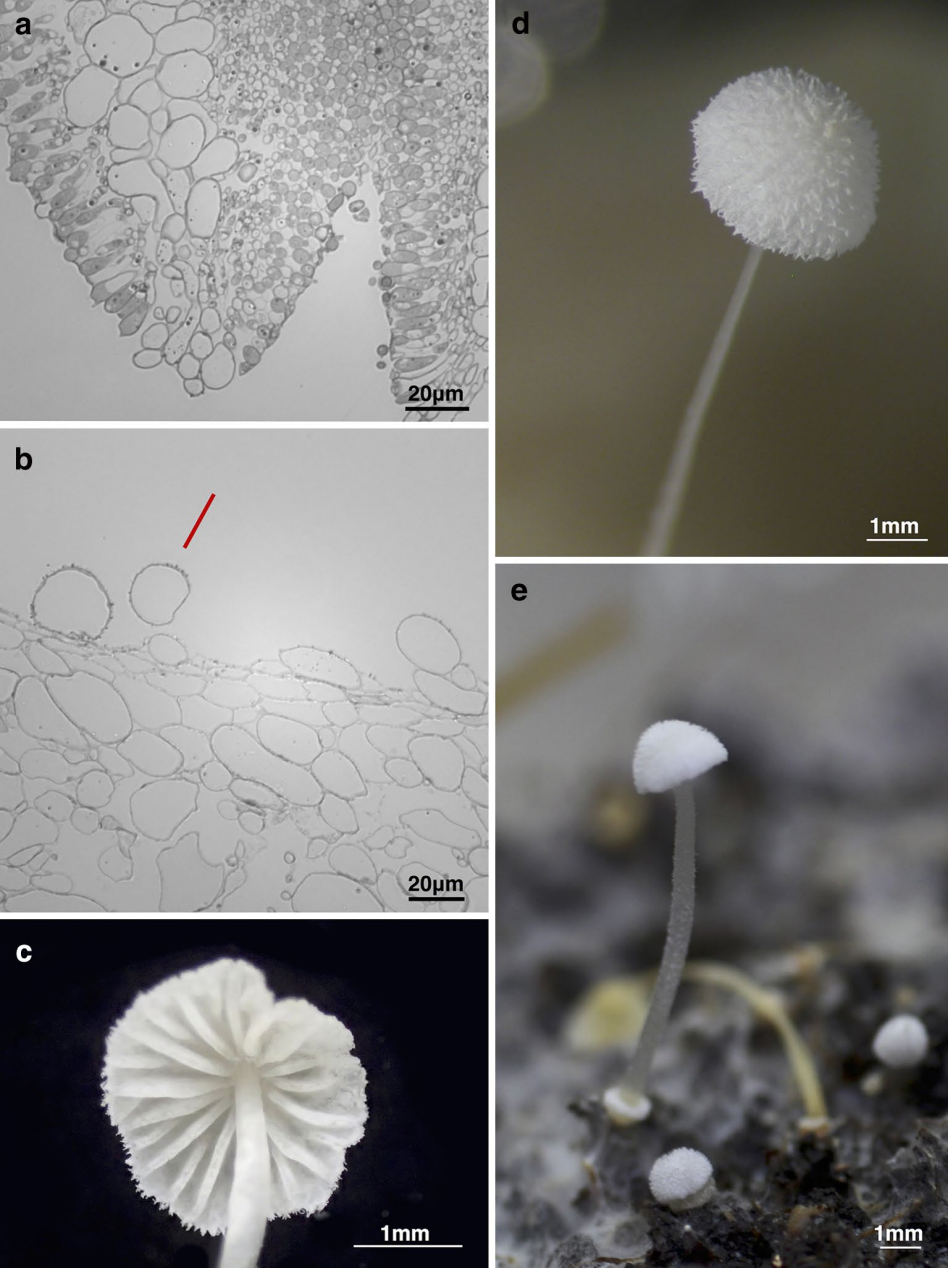
*Mycena albopilosa.* **a** Section of a gill. **b** Section of the pileus surface with the *red bar* indicating an acanthocysts. **c** Arrangement of gills. **d** Pileus of a basidioma. **e** Basidiomata produced on commercial soil and black rice bran of 4:1 ratio

**Fig. 2:**
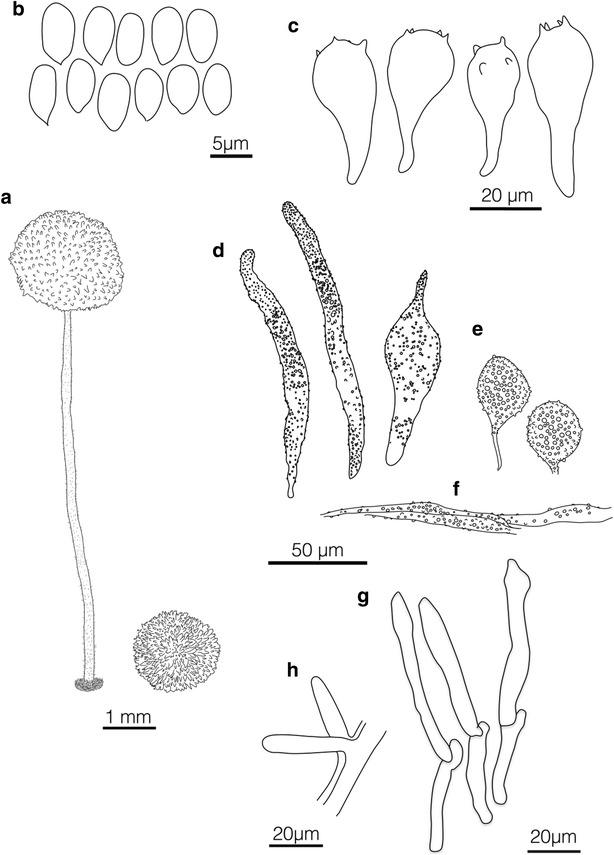
*Mycena albopilosa.* **a** Basidiomata and primordia overlain with conspicuous white hairs. **b** Basidiospores. **c** Basidia. **d** Pileocystidia. **e** Acanthocysts on pileus. **f** Pileipellis. **g** Cystidia on the basal disc. **h** Caulocystidia

**Fig. 3:**
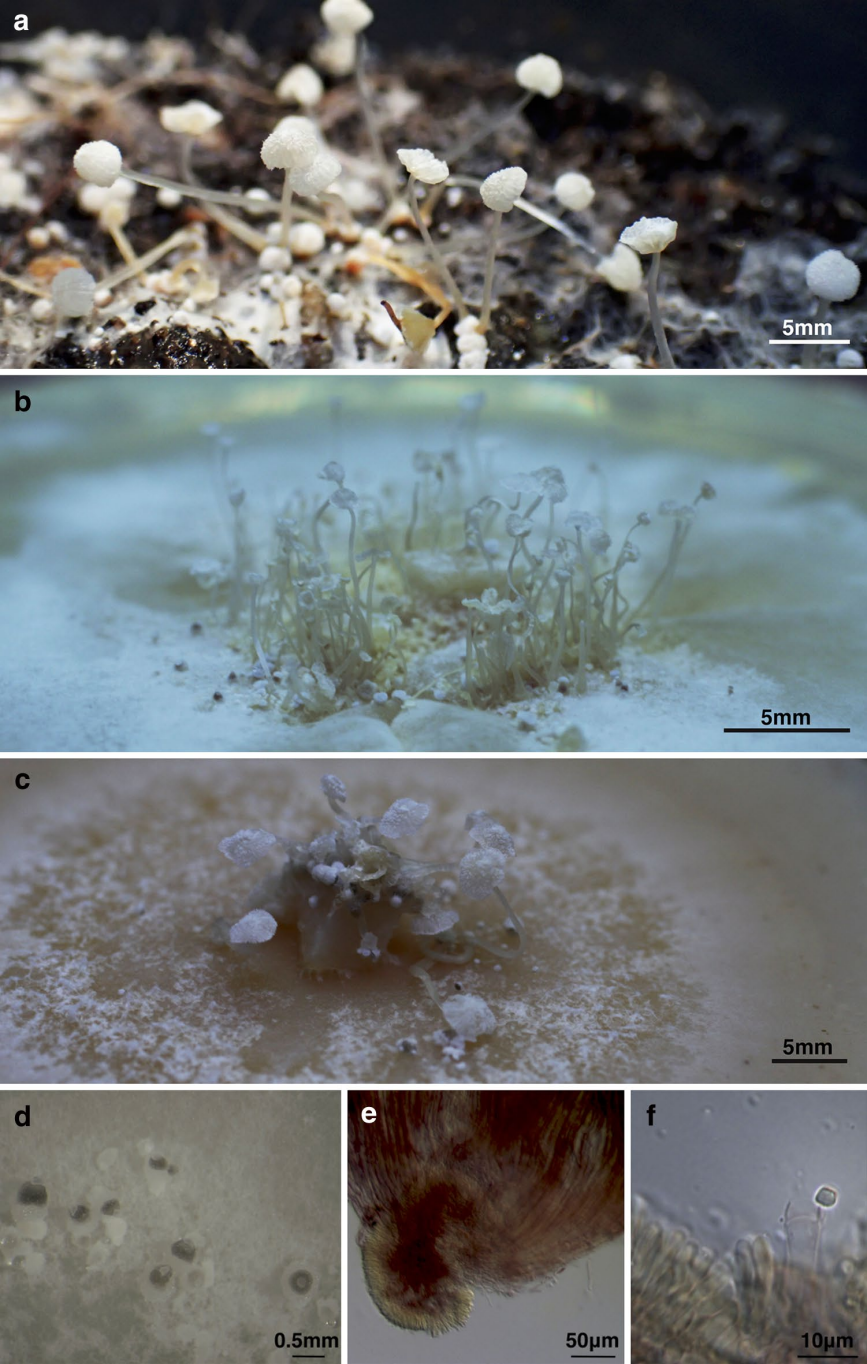
Basidiomata production of *Mycena albopilosa*. **a–c ** On commercial soil and black rice bran of 4:1 ratio, PDA, and OA, respectively. **d** Partially developed basidiomata on PDA. **e, f** Basidia and basidiospores produced on partially developed basidiomata

Etymology: referring to the white hairs on the pileate surface.

*Basidiomata* gregarious. *Pileus* pure white, bell-shaped, convex to flattened, 1.5–4 mm diam, sometimes sulcate-striate, densely covered with tufts composed of agglutinated pileocystidia and acanthocysts, faded as aged; margin entire or crenate, incurved as aged, with fragile flesh. *Pileipellis* cutis, smooth to verrucose on the hyphal wall. *Pileocystidia* cylindrical, lageniform to lanceolate, sometimes rostrate, 57–180 × 10–34 μm, warty, unevenly distributed. *Acanthocysts* abundant, globose to pyriform, 20–23 μm diam, warty, emerged from pileipellis hyphae. *Lamellae* adnexed, white, alternately arranged with lamellula; *trama* dextrinoid, composed of large globular to subglobular cells. *Basidia* hyaline, subclavate to pyriform, tetrasporic, 22–23 × 9–10 μm, inamyloid, thin-walled; *sterigmata* cone-shaped, 3.1–3.8 μm long, 1.5–1.7 μm at base. *Basidiospores* hyaline, ellipsoid to ovoid, (5–)5.5–7(–7.5) × (3.5–)4–4.7(–5.5) μm, Q = 1.2–1.76, smooth, inamyloid. *Basidioles* present. *Pleurocystidia* absent*. Cheilocystidia* absent. *Stipe* central, white to translucent, 7–15 × 0.4–0.7 mm, filiform, overlain with unevenly distributed caulocystidia, rising from a basal disc originated from remnant of primordia; *trama* turning cream (4A3) when aged or pressed, composed of cylindrical cells tightly and spirally arranged, septate, dextrinoid. *Caulocystidia* cylindrical, obtuse, sometimes clavate, smooth, 24–89 × 5–9 μm, single or 2–6 clustered. The basal disc remaining conspicuous through all growing stages, cup-shaped, 0.8–1.25 × 0.4–0.7 mm, floccose, composed of 4 or more chained cells, smooth, cylindrical to clavate, sometimes subglobose, obtuse to subacute, (19.5–)40–65(–70) × (5.2–)6–9(–14.2) μm, terminal cell sometimes rostrate, branched or warted. *Primordia* oblate to spherical, 0.75–2 mm × 0.25–0.5 mm tall, also covered with chalky white tufts of pileocystidia. *Clamp connections* readily seen in primordia remnant. *Smell* indistinctive.

#### Cultures and anamorph

*Colonies on formulated soil* covering Petri plates at 4 weeks, tomentose, white; basidiomata produced in 4 weeks.

*Colonies on PDA* 20 mm at 1 week, 32 mm at 2 weeks, 45 mm at 3 weeks, white to cream (4A3), irregular, wrinkled, wooly to tomentose, with powdered patches in places, sometimes radially sulcated and creased, with diffuse margins slightly fimbriate to tentacle-like and submerged; aerial hyphae inconspicuous; advancing zone 1.7–2.3 μm, sometimes 3–5 hyphae aggregated into bundles, aseptate; submerged hyphae branched, with globular enlargement; clamp connections abundant; odor indistinctive. *Basidiomata* produced in 18 days, much as those produced on formulated soil but differing in being extremely crowded, more variable in shape and generally smaller 1–1.5 mm diam; partially developed basidiomata sometimes produced.

*Colonies on MEA* 11 mm at 1 week, 20 mm at 2 weeks, 31 mm at 3 weeks, whitish, plane and waxy, with wavy margins; microscopic features as those on PDA.

*Colonies on OA* 20 mm at 1 week, 40 mm at 2 weeks, 55 mm at 3 weeks, irregular, partly floccose, with aerial hyphae brush-like, 0.5–1 mm long × 0.1–0.3 mm broad, white, with entire margins; basidiomata sometimes produced in 3 weeks, 3–6 mm diam, with macroscopic and microscopic features much as those produced on formulated soil; some primordia lacking further development, ball-shaped, loose and floccose, 0.20–0.65 diam.

#### Specimens examined

TAIWAN. New Taipei City, Shih-ting district, on dead fern frond on floor of broadleaf forest, 28 Feb 2014, Chang, Y.-Y. (dried culture, HOLOTYPE, HAST 142292), the holotype material composed of basidiomata produced on formulated soil, which are much like those collected from natural substrate, because the material collected from natural substrate was used up during the study.

#### Commentary

The salient features of *M. albopilosa* are the agglutinated pileocystidia resembling animal’s fur, conspicuous cup-like basal disc, lack of cheilocystidia, and inamyloid basidiospores. *Mycena albopilosa* is capable of producing abundant basidiomata on PDA within a short period of time, which may be proven useful for developmental studies of basidiomata. *Mycena albopilosa* can be placed in section *Sacchariferae*, which is characterized by a granular to floccose pileus surface and ascending lamellae (Maas Geesteranus 1992), and in stirps *Adscendens* Desjardin (1995), which possesses smooth caulocystidia but lacks cherocytes.

Twelve taxa have been placed in stirps *Adscendens* thus far. Based on the entirely smooth caulocystidia, *M. albopilosa* most closely resembles *M*. *adscendens* (Lasch) Maas Geesteranus var. *adscendens*, *M. adscendens* var. *carpophila* (J. E. Lange) Desjardin, and *M. cryptomeriicola* Imazeki & Toki. *Mycena albopilosa* differs from the typical variety and var. *carpophila* of *M. adscendens* in having a fairly floccose pileate surface rather than a somewhat granulose surface and smaller basidiospores. Cheilocystidia are absent in *M. albopilosa* but appear rostrate in the two varieties of *M*. *adscendens*. Bisporic basidia have never been observed in *M. albopilosa* but are commonly encountered in *M. adscendens* var. *adscendens* (Maas Geesteranus 1992; Desjardin 1995; Aronsen and Læssøe 2016). Furthermore, basidiospores are inamyloid in *M. albopilosa* but amyloid in the two varieties of *M. adscendens* in most cases. *Mycena cryptomeriicola* Imazeki and Toki (1955) was originally described as having inamyloid basidiospores in the section *Sacchariferae*. However, the holotype specimen is in poor condition, and recollected specimens were reported to have amyloid basidiospores (Tanaka and Hongo 2003). *Mycena albopilosa* differs from *M. cryptomeriicola* in mostly lacking ventricose-rostrate cheilocystidia.

*Mycena alphitophora* (Berk.) Sacc. and its varieties have floccose pilei and thus resemble *M. albopilosa* in gross morphology. However, *Mycena albopilosa* possesses a basal disc at all stages, whereas *M. alphitophora* and its varieties lack a basal disc but instead have a swollen stem base covered with wooly hyphae. In addition, *M*. *albopilosa* differs *M*. *alphitophora* and its varieties in lacking cheilocystidia and having smooth rather than spinulose caulocystidia (Table 1).

**Table 1:** Comparisons of *M. albopilosa* with similar species

Taxa	Basidiospores	Basidiospores per basidium	Cheilocystidia	Caulocystidia	Basal disc or bulb	Notes
*M. albopilosa*	Ellipsoid to ovoid, 5.5–7 × 4–4.7 μm, inamyloid	4	Absent	Smooth	Present	See the current study
*M. adscendens* var. *adscendens*	Ellipsoid, 8.3–10.2 × 5–6 μm, amyloid	2 (or 4)	Spinulose and rostrate	Smooth	Present	See Desjardin (1995)
*M. adscendens* var. *carpophila*	Ellipsoid, 8.3–10.2 × 4–4.5 μm, amyloid	4	Spinulose and rostrate	Smooth	Present	See Desjardin (1995); fruiting only on pericarps of *Fagus*
*M. alphitophora* var. *alphitophora*	Ellipsoid, 7.5–10 × 4.5–5.5 μm	4	Slightly spinulose, clamped	Spinulose	Absent	See Desjardin (1995); growing on fern debris
*M. alphitophora* var. *globispora*	Pyriform, subglobose or globose, 7–9 × 6–8 μm	4	Verrucose on upper half	Spinulose	Absent	See Manimohan & Leelavathy (1989)
*M. alphitophora* var. *distinda*	Pyriform, subglobose or globose, 6–8 × 3–4 μm	Mainly 2	Verrucose	Spinulose	Absent	See Manimohan and Leelavathy (1989); acanthocysts spinulose and rostrate
*M. cryptomeriicola*	Ellipsoid, 7–9 × 4–5 μm, inamyloid	4	Ventricose with 1–2 rostrae on top	Smooth	Present	See Desjardin (1995); hyphae unclamped; fruiting on coniferous leaves
*M. taiwanensis*	Broadly ellipsoid to pyriform, 7–9 × 4.5–6 μm	4	Verrucose	Smooth	Present	See Rexer (1994). Pileus color brownish

*Mycena albopilosa* produces basidiomata in culture easily. In contrast to the basidiomata produced in nature, those formed in culture tend to be more variable in shape, but a great portion of them are normal and fully developed. For those deviating basidiomata, agglutinated hyphal tufts on the pileus and primordia, basal discs, and microscopic characteristics remain the same, but the basidiomata are smaller in diameter (1–1.5 mm) and more fragile. Malformed characters of the basidiomata produced on PDA include partially developed to undeveloped pilei with a wide size range and twisted, smoother stipes. Although primordia are generally normal in microscopic features, they are three to four times smaller than those produced on formulated soil. It should be noticed that a translucent, circular hymenium was found forming directly on the shortened, globular stipe with blackened upper half on OA and PDA. The deformed stipe shows the same tissue construction as the normal stipe and is dextrinoid. The hymenium is inamyloid and composed of only basidia and basidioles. This phenomenon has never been found in other agarics.

An NCBI MEGABLAST query with the ITS sequence of *M*. *albopilosa* did not show high similarities with the top matches. The top 10 BLAST matches closest to *M. albopilosa* did not bear a species epithet. The next match with a known identity was KF007948, deposited by Baird, R. E. as *M. pura* (query coverage = 100%, identities = 589/691 [85%], gaps = 51/691 [7%]), which did not share a high identity with *M*. *albopilosa*. *Mycena pura* is a significantly different species from *M*. *albopilosa* in having a large pileus size of 20–50 mm diam with a pale pink color, having amyloid spores, and lacking pileocystidia and acanthocysts.

### *Gloiocephala epiphylla* Massee, Grevillea 21: 34. 1892., Fig. 4

*Basidiomata* peltate. *Pileus* flat, sunken as aged, rounded, sometimes irregular, 0.3–0.85 mm diam × 0.07–0.16 mm thick, white, turning pale orange (5A4) when aged, sometimes with brown spots at center. *Pileipellis* composed of enlarged globular to subglobular cells, 13–25.5 × 8.6–16.2 μm, scattered with pileogloeocystidia. *Pileogloeocystidia* capitate, 32–66 × 9–11 μm, with capitulum up to 19+ μm, transparent, becoming caramel (6C6-8); exudates hyaline, sticky, turning dark purple (14F8) as aged. *Flesh* consisted of small and irregular cells. *Hymenium* descending, white, powder-like and no gills formed. *Basidia* clavate, 19–22 × 6–7 μm, tetrasporic, hyaline, inamyloid. *Basidioles* present, the same shape as basidia. *Sterigmata* horn-shaped, 3.5–4.2 μm. *Cystidioles* clavate to lanceolate, 20–36 μm. *Basidiospores* ellipsoid to fusoid, inequilateral, (7.7) 8.4–10.2 (10.6) × (2.5) 3.2–3.9 (4.1) μm, Q = 2.4–3.1, hyaline. *Stipe* central to slightly eccentric, cylindrical, 2–2.8 × 0.5–0.7 mm, covered with gloeocystidia similar to those on pileipellis, white near the pileus and grading to light yellow (4A3–5) and then brown (6A8–6F8) towards the base.

**Fig. 4:**
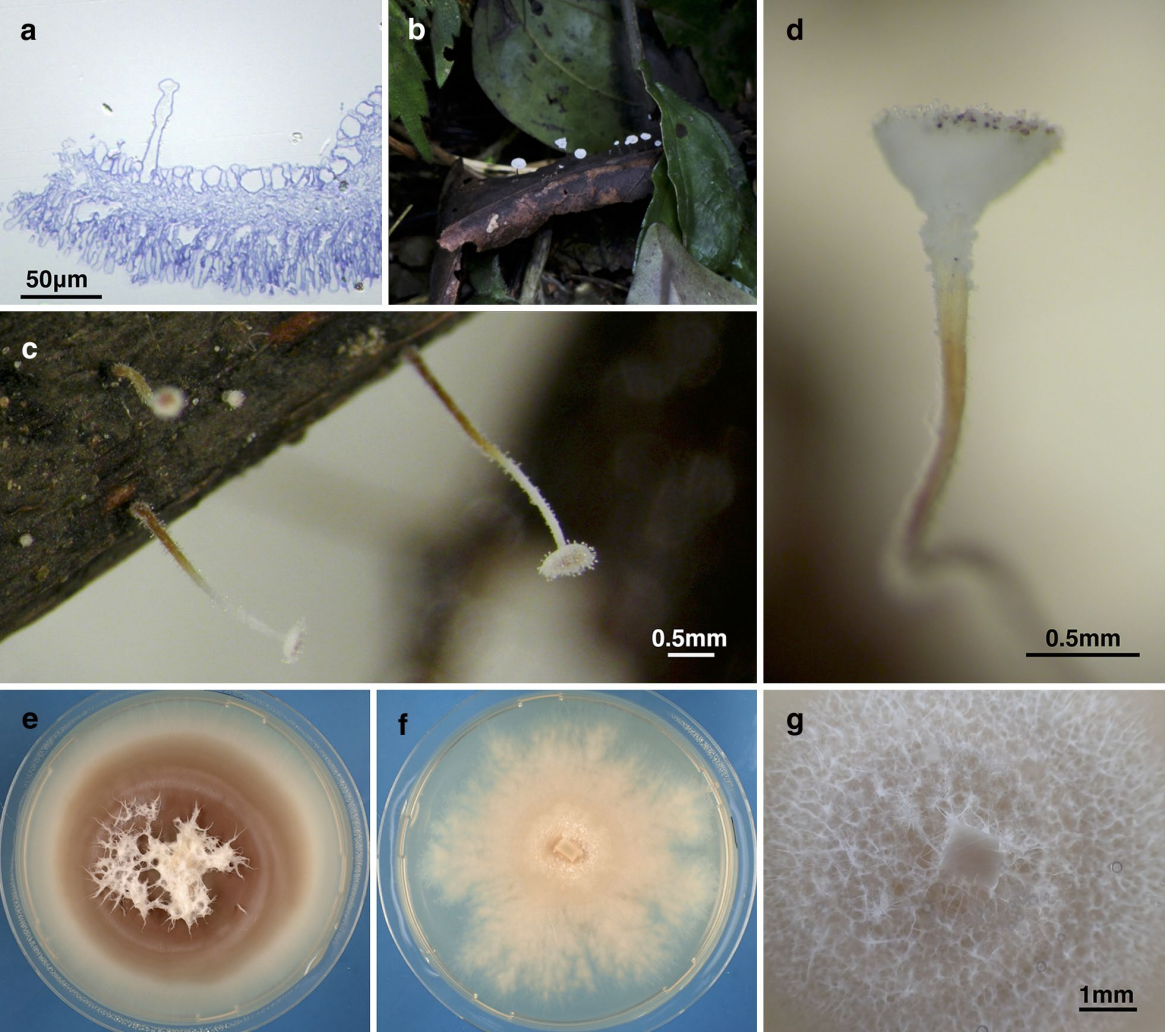
*Gloiocephala epiphylla.* **a** Section of pileus showing distinct layers and pileogloeocystidum. **b, c ** Basidiomata in natural habit. **d** Basidioma in detail. **e** Culture on PDA. **f** Culture on MEA. **g** Culture on OA

#### Cultures and anamorph

*Colonies on soil* (commercial soil and black rice bran of 4:1 ratio) full at 3 weeks, white, silk-like; basidiomata not produced.

*Colonies on PDA* 35 mm at 1 week, 70 mm at 2 weeks, full at 3 weeks, irregularly wrinkled, cream (4A3), becoming brown towards center, forming concentric zones of brown shade (7E4-8), occasionally scattered with viscid brown patches and dark brown spots (7F6-8), with submerged, diffuse margins; soluble pigment brown; aerial hyphae conspicuous, acicular, white, mainly distributed near center, bundled, branched. Aerial hyphae variable in diameter, twisted, smooth-walled, septate, sometimes interwoven and forming a thin membrane; submerged hyphae 1.5–2. 5 um diam, twisted, with globose enlargement (mostly at the end of hyphae), rarely septate; advancing zone septate, with short branches, obtuse at tip, 2.2–3.1 μm diam; clamp connections absent; odor strong, resembling burned plastic.

*Colonies on MEA* 33.5 mm at 1 week, 52.5 mm at 2 weeks, full at 3 weeks, plane, cream (4A3) to pinkish white, forming inconspicuous concentric rings, with irregular, plumose margins; aerial hyphae inconspicuous; microscopic features as those on PDA.

*Colonies on OA* 35 mm at 1 week, 50 mm at 2 weeks, full at 3 weeks, hyaline to white, with entire margins, covered with conspicuous white, evenly distributed aerial hyphae; microscopic features as those on PDA.

#### *Specimen examined*

TAIWAN. New Taipei City, Wulai, Red River Valley, elev. ca. 100 m, scattered on decayed broad leaves, 2 Apr 2014, Chang, Y.-Y. (HAST 142293).

#### Commentary

*Gloiocephala epiphylla*, the type species of the genus (Singer 1976), was originally described growing on damp, decaying leaves from Jamaica in Massee (1892), where monosporic basidia and globose basidiospores were recorded. However, specimens collected in Ecuador, Japan and Hawaii have tetrasporic basidia (Desjardin et al. 1992), with which our Taiwan collection agrees. *Gloiocephala epiphylla* is widely distributed, being reported from Jamaica, Puerto Rico, Venezuela, Ecuador, Argentina, Japan, Hawaii (Desjardin et al. 1992), tropical Africa (Antonín 2007), and Taiwan. Features of our Taiwan collection correspond well with those described by Desjardin et al. (1992) and Antonín (2007) except for the pileus size being smaller, i.e., 0.3–0.85 mm diam vs. 1.5–4 mm diam.

*Gloiocephala epiphylla* is a newly recorded genus and species in Taiwan, being characterized by lamellae reduced or lacking, basidiospores inamyloid, and possession of secretory pileogloeocystidia (Antonín 2007). The secretory pileogloeocystidia on the basidiomata are so conspicuous that they appear spine-like by hand lenses. In Taiwan, the basidiomata were found growing on fallen leaves, twigs or decaying fruits in low-latitude broadleaf forests in spring. Desjardin et al. (1992) recorded that *G. epiphylla* were commonly found along the coastal areas or watercourses. Our collection in Taiwan was made from upper bank forest floor. Nevertheless, the collecting site is humid, and the forest floor is constantly flooded by temporary water flow from the rain fall.

An NCBI MEGABLAST query by using ITS sequence from the Taiwan collection matched well DQ097357 of *G. epiphylla* deposited by Binder et al. (2006) (query coverage = 100%, identities = 770/774 [99%], gaps = 0/774 [0%]).

## Key to *Mycena* species reported in Taiwan

A list of *Mycena* species known in Taiwan can be found in *Catalogue of life in Taiwan* (Shao 2009), to which references to the listed species are referred. Five of the species lack a local description and are denoted with an asterisk (*). *Mycena photogena* Kominami is an invalidly published name, which appears in Sawada (1933) and is excluded from this key. The key was adapted mainly from descriptions in Maas Geesteranus (1992), Desjardin (1995) and Aronsen and Læssøe (2016).

1. Pileus viscid; hyphae of pileipellis forming a gelatinous, separable layer……2
2. Pileus dry or moist, in some cases becoming lubricous; pileipellis not separable as a gelatinous layer……6
  2. Basidiomata fairly small; pileus rarely exceeding 10 mm; stipe rising from a conspicuous basal disc……3
  2. Basidiomata medium-sized to large; pileus up to 30 mm; stipe lacking a basal disc……5
3. Cheilocystidia covered with short, even-length excrescences (warts), broadly clavate to subglobose; caulocystidia widely ellipsoid to subglobular, sometimes rostrate
  ……*M. taiwanensis* Rexer (Rexer 1994)
  [*Mycena taiwanensis* is not listed in Shao (2009). Although *M*. *taiwanensis* was described in a Ph.D. dissertation (Rexer 1994), according to *International Code of Nomenclature for algae, fungi, and plants* (*Melbourne Code*) Article 30.8 & Ex. 17, it is considered as a validly published name because the dissertation was effectively published by the German commercial printer Zeeb-Druck]
4. Cheilocystidia smooth, branched, apically narrowed; caulocystidia fusiform……4
  4. Pileus depressed at center, smooth, luminescent at pileus and stipe; stipe puberulous; pileocystidia present
    ……*M. chlorophos* (Berk. & Curt.) Sacc. (Maas Geesteranus 1992)
  4. Pileus not depressed at center, with a few spinules, luminescent at mycelium only; stipe glabrous except at the base; pileocystidia absent……*M. stylobates* (Pers.) P. Kumm. (Maas Geesteranus 1992)
5. Basidiomata reddish orange; lamellar edge not separable as an elastic-tough thread; cheilocystidia clavate, fusiform or forked……*M. leaiana* (Berk.) Sacc. (Maas Geesteranus 1992, Chou and Wang 2004)
6. Basidiomata brown to pale grey-brown; lamellar edge separable as an elastic-tough thread; cheilocystidia profusely branched……*M. vulgaris* (Pers.) P. Kumm. (Maas Geesteranus 1992)
  6. Pileus floccose or granular on surface; lamellae ascending; stipe hairy, with base or basal disc; cheilocystidia clavate to fusiform, covered with short, even-length excrescences (warts)……7 (sect. *Sacchariferae*)
  6. Pileus glabrous on surface……10
7. Stipe base lacking a disc or bulb, rather broadened and covered with white wooly hair; caulocystidia spinulose……*M. alphitophora* (Berk.) Sacc. (Desjardin 1995)
  [Liou (1980) recorded this fungus as *M. osmundicola* J. E. Lange, which is treated as a synonym of *M*. *alphitophora* in Desjardin (1995)]
8. Stipe base with a basal disc or bulb; caulocystidia if present, smooth……8
  8. Pileus extremely floccose; pleurocystidia present; cheilocystidia absent; basidiospores inamyloid……***M. albopilosa***
  8. Pileus granulose; pleurocystidia absent; cheilocystidia distinctively spinulose; basidiospores mostly amyloid……9
9. Pileus luminescent; caulocystidia absent; basidia tetrasporic
  ……*M. kentingensis* Y.S. Shih, C.Y. Chen, W.W. Lin and H.W. Kao (Shih et al. 2014)
10. Pileus not luminescent; caulocystidia present; basidia mostly bisporic……*M. adscendens* Maas Geest. (Desjardin 1995)
  10. Stipe exuding blood red fluid when cut……*M. haematopus* (Pers.) P. Kumm. (Maas Geesteranus 1992)
  10. Stipe not exuding blood red fluid when cut……11
11. Lamellar edge more intensely colored than lamellar sides……12
12. Lamellar edge concolorous with or in a similar color to lamellar sides……13
  12. Strong nitrous smell; lamellar sides with densely distributed red–brown dots……M. capillaripes* (Maas Geesteranus 1992)
  12. Smell indistinct; lamellar sides lacking red–brown dots……*M. roseomarginata* Hongo (Maas Geesteranus 1992)
13. Basidiospores inamyloid; pileus color bright orange–red……*M. adonis* (Bull.: Fr.) S. F. Gray (Maas Geesteranus 1992)
14. Basidiospores amyloid; pileus color whitish or brownish……14
  14. Hyphae of the pileipellis, caulocystidia, and cheilocystidia smooth……15
  14. Hyphae of the pileipellis, caulocystidia, or cheilocystidia warted, with excrescences or branched……16
15. Basidiomata cespitose; odor pungent, yeast-like……*M. overholtsii* A.H. Smith and Solheim* (Maas Geesteranus 1992)
  [*Mycena overholtsii* is occasionally misspelled as *M. overholtsii.* Both names are listed in Shao (2009)]
16. Basidiomata scattered; odor raphanoid……*M. pura* (Pers.: Fr.) Kumm.* (Maas Geesteranus 1992)
  16. Pileus often less than 2.5 mm; stipe arising from a whorl of radiating dark-brown hyphae……*M. capillaris* (Schum.: Fr.) Fr. (Maas Geesteranus 1992)
  16. Pileus often larger than 5 mm; stipe not arising from a whorl of radiating dark-brown hyphae……17
17. Lamellar edge homogenous, with cheilocystidia forming a continuous sterile band……18
18. Lamellar edge heterogeneous, with cheilocystidia mixed with basidia, forming a discontinuous band……20
  18. Basidiomata usually fasciculate; odor strong when cut; cheilocystidia covered with unevenly distributed excrescences……19
  18. Basidiomata usually solitary or in small groups; odor indistinct; cheilocystidia mostly covered with evenly distributed excrescences……*M. filopes* (Bull.) P. Kumm. (Maas Geesteranus 1992, Chou and Wang 2004)
    [Chou and Wang (2004) recorded this fungus as *M. amygdalina*, which is listed as a synonym of *M. filopes* in *Species Fungorum* (Kirk 2016)]
19. Pileus margin spotted with vinaceous stains; the stipe not forming a rooting base; pileipellis sparsely diverticulate, not embedded in gelatinous matter……*M. inclinata* (Fr.) Quèl. (Maas Geesteranus 1992)
20. Pileus margin not spotted, usually upturned when mature; the stipe forming a rooting base; pileipellis covered with densely branched excrescences, embedded in gelatinous matter……*M. polygramma* (Bull.: Fr.) S. F. Gray (Maas Geesteranus 1992, Chou and Wang 2004)
  20. Pleurocystidia numerous; pileipellis simple to highly branched; cheilocystidia fusiform to clavate, unevenly covered with short excrescences
    ……*M. latifolia* (Peck) A. H. Smith (Maas Geesteranus 1992, Chou and Wang 2004)
  20. Pleurocystidia absent; pileipellis the narrower smooth, the wider sparsely warted or diverticulated; cheilocystidia clavate to irregular in shape, covered with a few to many long excrescences that are simple or highly branched……*M. maculata* P. Karst.* (Maas Geesteranus 1992)
    [Shao (2009) adopted the name *M. alcalina* (Fr.) P. Kumm., with which *M. maculata* is considered in synonymy. However, Maas Geesteranus (1992) recognized them as two distinct species and stated that *M. maculata* is often misapplied to *M. alcalina*]
